# Acute Mental Disorder Caused by Vitamin B12 Deficiency Eight Years After Total Gastrectomy

**DOI:** 10.7759/cureus.68507

**Published:** 2024-09-03

**Authors:** Takayoshi Tsubaki, Mitsuhiro Morikawa, Takanori Goi, Yasuo Hirono

**Affiliations:** 1 Cancer Care Promotion Center, University of Fukui Hospital, Fukui, JPN; 2 Department of First Surgery, Faculty of Medicine, University of Fukui, Fukui, JPN

**Keywords:** intrinsic factor deficiency, iron deficiency, mixed anemia, macrocytic anemia, orthoblastic anemia, total gastrectomy, vitamin b12 deficiency

## Abstract

Vitamin B12 deficiency can cause a variety of diseases. The most common disease is macrocytic anemia, but it has also been found to be a cause of psychiatric disorders. The causes of deficiency are varied, and diagnosis is often difficult. Here, we report a patient who developed mental disorders due to vitamin B12 deficiency after total gastrectomy.

A 37-year-old female, eight years after total gastrectomy, was withdrawn at her workplace, talking and acting abnormally. The family had seen unusual behavior for three days. The patient had no particular history of mental illness. The possibility of herpes encephalitis was suspected, and the patient was referred to our hospital, but there were no specific findings in the head on imaging examination. Blood tests showed no macrocytic anemia. Spinal fluid cytology and electroencephalography showed no specific findings, and herpes DNA was negative. Metabolic factors such as vitamin deficiency were considered, and intravenous vitamin replacement therapy was initiated. The psychiatric symptoms improved rapidly after vitamin B12 supplementation was started. On the fifth day of her hospitalization, it was discovered that her vitamin B12 level at the time of admission was extremely low. Typically, vitamin B12 deficiency is associated with macrocytic anemia, but in this patient, serum iron was also decreased, indicating a mixed anemia, making the diagnosis difficult. The patient had undergone a total gastrectomy for gastric cancer eight years ago, and the psychiatric symptoms were thought to be due to impaired vitamin B12 absorption caused by intrinsic factor deficiency. Since then, oral replacement therapy and intramuscular injection have been continued without recurrence of symptoms.

Disturbances of consciousness may have many causes, but when there is a history of gastrectomy, we should also consider vitamin B12 deficiency when examining patients.

## Introduction

There are many vitamins that support the vital activities of the human body, and vitamin B12 is one of them. Vitamin B12 is found only in foods of animal origin, such as liver, beef, chicken, eggs, and dairy products [1,2]. It is bound to proteins in food but is cleaved from the proteins by hydrochloric acid secreted from the gastric mucosa and absorbed from the distal ileum after forming a complex with endogenous factors [2]. The average adult's total body vitamin B12 stores are said to be 0.6-3.9 mg, and deficiency causes a variety of symptoms [1]. Typical symptoms include megaloblastic anemia, peripheral neuropathy, and psychiatric disorders such as depression, mania, and acute psychosis [3-5]. The recommended dietary allowance (RDA) is 2.4 μg for adult men and non-pregnant women and 2.6 μg for pregnant women [5]. Causes of deficiency include low vitamin B12 intake, malabsorption, increased requirements, and unbalanced diets [3,4,6]. Endogenous factor deficiency after total gastrectomy also leads to decreased absorption in the body and is one of the causes. Vitamin B12 supplementation is necessary after total gastrectomy, but if inadequate, symptoms appear when body stores are depleted.

The patient developed psychiatric symptoms eight years postoperatively due to inadequate replacement therapy after total gastrectomy. Vitamin B12 is involved in DNA synthesis and red blood cell production, so a deficiency increases mean corpuscular volume (MCV) due to altered red blood cell production, usually resulting in macrocytic anemia [7]. However, since serum iron was also deficient in this case, the patient presented with mixed anemia, making the diagnosis difficult. Patients who have undergone total gastrectomy should be supplemented with vitamin B12, but iron supplementation should also be given at the same time, with periodic monitoring of blood tests for values.

## Case presentation

A 37-year-old female was found holed up in the office of her workplace. She exhibited disorientation and did not leave the place for two hours. For the past three days, she had been laughing at trivial things, which was unusual even for her family. The patient was referred to our hospital because herpes encephalitis was suspected due to the acute onset of psychiatric symptoms. At the time of her visit, her consciousness was somewhat hazy, but she maintained her disorientation. The patient was admitted to the hospital for follow-up, although there were no obvious initial neurological findings. Her past medical history was a total gastrectomy for gastric cancer eight years ago. Three years and four months postoperatively, the patient had a recurrence with positive peritoneal lavage cytology and lymph node metastasis in the right hilar region. However, intraperitoneal chemotherapy and chemoradiotherapy were successful, and she continued chemotherapy and remained recurrence-free. At the onset, it was 15 days after the 45th administration of docetaxel (40 mg/body) + cisplatin (40 mg/body) (bi-weekly).

She and her family had no history of mental illness. No developmental or growth abnormalities were ever noted. There were no regular medications other than 2 mg of dexamethasone taken during chemotherapy. On physical examination, the patient had a height of 157 cm, a body weight of 43 kg, a pulse of 112 beats/minute, a blood pressure of 105/62 mmHg, and a body temperature of 37.3℃. There was no edema, rash, or jaundice. The cervical lymph node and thyroid gland were not palpated. Respiratory sounds were clear, and there was no difference between the left and right lungs. Her abdomen was flat and soft, and there was no spontaneous pain or tenderness. Laboratory tests on the day of admission showed normocytic anemia (hemoglobin level: 8.5 g/dL, mean corpuscular volume level: 81.4 fL). There were no electrolyte abnormalities, hypoglycemia, or elevated ammonia (Table 1). Vitamin B1 and B12 results were not available on admission. There were no abnormal findings in the cerebrospinal fluid examination. Spinal fluid cell count, protein, and sugar were all within normal limits. Herpes DNA also came back negative. Computed tomography (CT) and magnetic resonance imaging (MRI) of the head revealed no evidence of metastasis, inflammation, or edema. Single-photon emission computed tomography (SPECT) of the brain showed no increased or decreased blood flow.

**Table 1:** Results of blood sampling at admission MCV: mean corpuscular volume, CRP: C-reactive protein, BUN: blood urea nitrogen, AST: aspartate aminotransferase, ALT: alanine aminotransferase

Laboratory test	Actual result	Normal range
White blood cell count	3.2×10^3^/μL	3.3-8.6×10^3^/μL
Hemoglobin	8.5 g/dL	11.6-14.8 g/dL
Platelet	296×10^3^/μL	158-348×10^3^/μL
MCV	81.4 μm^3^	83.6-98.2 μm^3^
CRP	<0.01 mg/dL	0-0.14 mg/dL
Natrium	142 mmol/L	138-145 mmol/L
Potassium	4 mmol/L	3.6-4.8 mmol/L
Chlorine	107 mmol/L	101-108 mmol/L
Calcium	9.2 mg/dL	8.8-10.1 mg/dL
Total protein	7 g/dL	6.6-8.1 g/dL
Albumin	4.1 g/dL	4.1-5.1 g/dL
Cholinesterase	266 U/L	201-421 U/L
BUN	22 mg/mL	8-20 mg/mL
Creatinine	0.84 mg/mL	0.46-0.79 mg/mL
AST	22 U/L	13-30 U/L
ALT	15 U/L	7-23 U/L
Serum iron	16 μg/dL	40-188 μg/dL
Glucose	101 mg/mL	73-109 mg/mL
Ammonia	30 μmol/L	30-130 μmol/L

Symptoms temporarily resolved after hospitalization. However, on the second day of hospitalization, the patient was seen laughing alone in her hospital room, and it was suspected that she was suffering from a recurrence of symptoms. Metabolic factors such as vitamin deficiency and epilepsy were considered, and for that reason, intravenous vitamin supplementation (100 mg/day of fursultiamine hydrochloride (vitamin B1 derivative preparation: ALINAMIN®︎-F 100), thiamine disulfide phosphate, pyridoxine hydrochloride (vitamin B6), and cyanocobalamin (vitamin B12) (VITAMEDIN®︎)) was started. On the third day of illness, she was depressed and tended to sleep in the morning. She did not eat at all and had difficulty in speaking. An electroencephalogram (EEG) showed no seizure waves suggestive of epilepsy. Acyclovir (250 mg three times/day) was continued until the herpes DNA results came back. From the fourth day of illness, the patient no longer laughed persistently, and communication became easy. On the fifth day of illness, the admission laboratory tests revealed very low vitamin B1 and B12 levels (Table 2). The patient was started on 1,500 µg/day of oral mecobalamin (vitamin B12 preparation: Methycobal®︎). On the sixth day of illness, the patient's communication was normal, and mental symptoms such as laughing alone disappeared. It was judged that the patient could be discharged.

**Table 2:** Vitamin B1 and B12 results at admission

Laboratory test	Actual result	Normal range
Vitamin B1	1.8 μg/mL	2.6-5.8 μg/mL
Vitamin B12	136 pg/mL	233-914 pg/mL

After discharge, the patient continued to take 1,500 μg/day of mecobalamin and 75 mg/day of fursultiamine hydrochloride (ALINAMIN®︎-F sugar-coated tablets). The first outpatient visit was on the 13th day after discharge, and there was no flare-up of symptoms by that time. Vitamin B1 had improved to 24.1 μg/mL and vitamin B12 to 805 pg/mL. Vitamin B12 returned to normal range. The patient continued to take vitamin B12 for the next five months, but it became too high (>1,500 pg/mL), so the medication was terminated.

**Figure 1:**
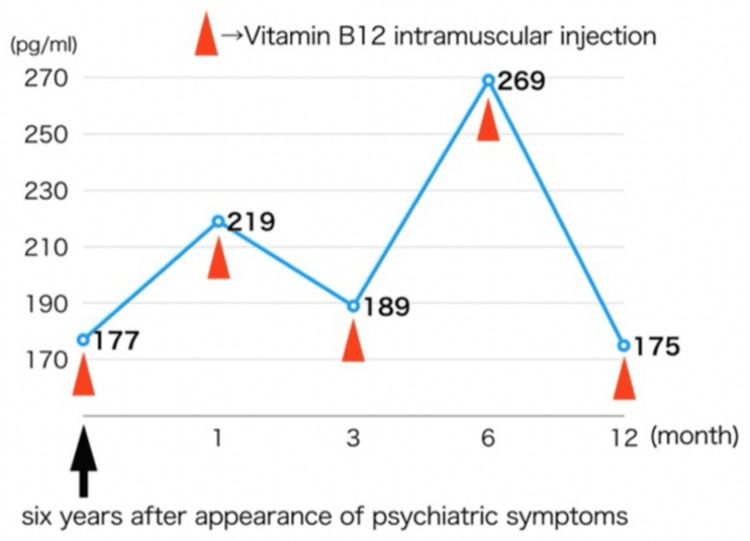
Vitamin B12 levels Six years had passed since the patient stopped taking the medication after the initial onset, so vitamin B12 was low again. Vitamin B12 was checked, and muscle injections were given during regular visits. The level has remained somewhat low.

Since then, the patient has been attending the outpatient clinic. However, six years later, when her vitamin B12 levels were retested, it was down to 177 pg/mL, so she was given a vitamin B12 intramuscular injection (500 μg of mecobalamin). She continues to receive the same amount of vitamin B12 intramuscular injections at regular visits (Figure 1). Vitamin B12 levels are unstable, but the patient is progressing without symptom flare-ups.

## Discussion

Vitamin B12 is one of the water-soluble vitamins that humans need to survive. It is found in meat, eggs, and dairy products, and most of which are stored in the liver (2-3 mg) [8]. The median daily intake of food in the United States is said to be about 5 μg for men and 3.5 μg for women [1]. Decreased vitamin B12 intake may result in clinical symptoms after 5-10 years [3]. Major causes of deficiency include decreased ileal absorption (Crohn's disease and ileal resection), decreased intrinsic factor (atrophic gastritis and post-gastrectomy), and inadequate intake (strict vegetarians, vegans, elderly, and alcoholics). Major clinical manifestations include skin disorders, glossitis, hematologic abnormalities, and neuropsychiatric abnormalities, such as dementia-like symptoms and memory loss, depression, and acute psychosis [4,5]. Hematologic abnormalities include anemia with macrocytic [4].

The patient had undergone total gastrectomy for advanced gastric cancer eight years ago and was intrinsic factor deficient. She did not receive adequate postoperative vitamin B12 supplementation. For this reason, the psychiatric symptoms are thought to have occurred when the body's stores of vitamin B12 were depleted. The fact that the symptoms were quickly relieved by the administration of a multivitamin containing vitamin B12 was also consistent with the characteristics of the disease.

Vitamin B12 deficiency is usually associated with macrocytic anemia, but this patient had normocytic anemia. She had not received any iron supplementation since the total gastrectomy, and serum iron was low at 16 μg/dL at the time of hospitalization. The patient also had iron deficiency anemia, which was difficult to diagnose because the patient had mixed anemia. Iron as well as vitamin B12 would need to be properly supplemented and monitored with regular blood tests.

Vitamin B1 was also low, but there was no ocular motility disorder or gait disturbance, so we consider that the patient did not have Wernicke's encephalopathy.

On the other hand, a limitation of this case report is the incomplete exclusion of delirium. A distinction should have been made between delirium caused by a medical illness and acute mental disorder for acute-onset psychiatric symptoms. Detailed observations of psychiatric symptom changes, such as continuity and diurnal variation, should have been made.

The treatment for vitamin B12 deficiency is either intramuscular injection of mecobalamin or oral supplementation. Vitamin B12 absorption essentially requires intrinsic factors, but previous reports have indicated that passive diffusion does not require intrinsic factors. The daily requirement can be met by taking high doses of vitamin B12 orally, even if the patient has undergone a total gastrectomy [9]. The required daily oral dose is approximately 1,000 μg [6]. In the present case, the patient continued to take 1,500 μg/day after discharge from the hospital, and the vitamin B12 level became very high, and the medication was discontinued. Six years after discontinuation, the level became low again, and she is now receiving regular intramuscular injections. However, the levels are not stable, so continued oral administration of 1,000 µg/day is being considered. In addition, iron supplementation is required to prevent iron deficiency anemia.

Vitamin B12 deficiency after total gastrectomy is often thought of as macrocytic anemia, but neurological symptoms can also occur, although they are rare [10,11]. However, a PubMed search using keywords such as "psychosis," "total gastrectomy," and "vitamin B12" did not yield any hits, suggesting that it is not well known. Therefore, clinicians must be aware of the possibility of psychiatric symptoms such as those seen in the present case in patients who have undergone total gastrectomy.

## Conclusions

Disturbances of consciousness are routinely encountered emergencies, and their causes are varied and often challenging to determine. This case underscores the importance of considering vitamin B12 deficiency as a potential cause of psychiatric symptoms, particularly in patients with a history of total gastrectomy. Vitamin B12 deficiency can present with neuropsychiatric manifestations even in the absence of macrocytic anemia, making the diagnosis challenging. Early recognition and treatment of vitamin B12 deficiency are crucial, as timely supplementation can lead to rapid improvement in psychiatric symptoms. This case highlights the need for continuous monitoring of vitamin B12 levels and appropriate supplementation in post-gastrectomy patients to prevent the recurrence of symptoms. Clinicians should maintain a high index of suspicion for vitamin deficiencies in similar clinical scenarios to ensure comprehensive patient care.
